# Physical fitness, cardiovascular and musculoskeletal health, and occupational performance in firefighters

**DOI:** 10.3389/fpubh.2023.1241250

**Published:** 2023-08-25

**Authors:** Jaron Ras, Denise L. Smith, Andre P. Kengne, Elpidoforos S. Soteriades, Lloyd Leach

**Affiliations:** ^1^Department of Sport, Recreation and Exercise Science, Faculty of Community and Health Sciences, University of the Western Cape, Cape Town, South Africa; ^2^Health and Human Physiological Sciences, Skidmore College, Saratoga Springs, NY, United States; ^3^Non-communicable Diseases Research Unit, South African Medical Research Council, Cape Town, South Africa; ^4^Healthcare Management Program, School of Economics and Management, Open University of Cyprus, Nicosia, Cyprus; ^5^Environmental and Occupational Medicine and Epidemiology (EOME), Department of Environmental Health, Harvard T.H. Chan School of Public Health, Boston, MA, United States

**Keywords:** firefighters, occupational performance, ability test, cardiovascular, musculoskeletal, physical fitness, cardiorespiratory, strength and endurance

## Abstract

**Introduction:**

To perform their work efficiently and safely, firefighters should maintain all aspects of physical fitness. Cardiac-related incidents are the leading cause of duty-related deaths in firefighters, and many firefighters have poor musculoskeletal health (MSH) that hinder their occupational performance (OP). Establishing the relationship between physical fitness, cardiovascular health (CVH), MSH and OP may add new insight on the most significant factors influencing OP in firefighters, specifically in the City of Cape Town Fire and Rescue Service (CoCTFS), which had not been studied before. Therefore, the purpose of this study was to investigate whether physical fitness, CVH and MSH were associated with OP in firefighters, in the COCTFRS.

**Methods:**

This cross-sectional study included 283 full-time firefighters aged 20–65 years from Cape Town, South Africa. A researcher-generated questionnaire was used to collect data on sociodemographic characteristics, lifestyle factors and MSH. Physical measures were used to collect information on physical fitness, CVH, and OP [using a physical ability test (PAT)]. Linear and binary logistic regressions, adjusted for age, sex, height and weekly metabolic equivalent minutes (WMETM), multivariate analysis of covariance (MANCOVA), adjusted for age, sex, height and body mass index (BMI) and backward stepwise regressions were used to investigate the associations between the various constructs.

**Results:**

From multivariable analyses, age, lean body mass, body fat percentage (BF%), estimated absolute oxygen consumption (abV̇O_2max_), grip strength, leg strength, push-ups, sit-ups, WMETM and heart rate variability were associated with PAT completion times (all *p* < 0.01). The MANCOVA showed a significant difference between performance categories of the PAT based on physical fitness and CVH (both *p* < 0.001). WMETM, BF%, abV̇O_2max_, grip strength, leg strength and sit-ups explained the highest proportion (50.5%) of the variation in PAT completion times.

**Conclusion:**

Younger, non-obese, fitter and stronger firefighters, with a better CVH status, performed significantly better and were most likely to pass the PAT in firefighters, in Cape Town, South Africa. Firefighters should maintain high levels of physical fitness and a good level of CVH to ensure a satisfactory level of OP.

## 1. Introduction

Firefighting is a strenuous occupation that involves routine exposure of firefighters to high temperatures, hazardous chemicals and fumes, which, along with the high physical demands, present a substantial burden on the cardiovascular system (1, 2). These exposures require firefighters to wear heavy, insulated personal protective equipment (PPE), all of which, place significant strain on their cardiovascular and musculoskeletal systems (3, 4). In addition, in order to perform their work efficiently and safely, firefighters are required to maintain all aspects of their physical fitness (5–7).

Previous studies have reported that several firefighting tasks have an average oxygen consumption (V̇O_2_) of 23.0 to 42.5 mL·kg^^−1^^·min^^−1^^ (8–10), with the most strenuous tasks requiring an average of 44.0 mL·kg^−1^·min^−1^ (11). In order to effectively handle these job demands, firefighters should maintain a cardiorespiratory fitness level of about 42 mL·kg^−1^·min^−1^ (3), while also being encouraged to maintain good levels of muscular strength and muscular stamina to perform their duties adequately (12). Firefighters who are unable to perform their intense duties with sufficient competency and efficiency are at risk of underperforming while on active duty (12–14). An inability to complete required job tasks in a timely manner not only places their lives at risk, but also the lives of the civilians, while also increasing the risk of potential damage to property and infrastructure (1, 12).

Due to the strenuous nature of firefighting, firefighters who are relatively unfit may have to overexert themselves to carry out their duties to an acceptable standard (1, 2). Furthermore, firefighters that have subclinical cardiovascular disease or an unfavourable cardiovascular disease (CVD) risk profile are particularly susceptible to cardiac incidents related to overexertion, which occur at an unacceptable rate (15, 16). In fact, cardiac-related incidents are the leading cause of duty-related deaths among firefighters, accounting for 40–50% of all line-of-duty firefighter fatalities in the United States. Many of these firefighters have underlying CVD risk factors, such as smoking, hypertension, dyslipidaemia, diabetes and obesity (1, 15–17). This is consistent with previous findings from a study conducted on firefighters in the City of Cape Town Fire and Rescue Service (CoCTFRS), where it has been reported that firefighters had multiple CVD risk factors, most notably being dyslipidaemia (40.3%), cigarette smoking (39.5%), obesity (37.1%), and hypertension (33.1%) (18). Firefighters in Cape Town have been reported to have a good knowledge of CVD risk factors, however, had poor attitudes toward health habits related to improve CVD risk, such as physical activity and diet, which become progressively worse as they age (19, 20). This has also been shown in previous studies, indicating that attitudes progressively become worse in firefighters throughout their careers, perhaps attributable to the stressful nature of the occupation (21–23). Moreover, the stressors of firefighting also contribute to work-related musculoskeletal injuries and musculoskeletal discomfort (24, 25). In one research study, firefighters reported that musculoskeletal pain negatively affected their work output and was associated with work limitations (26). Previous research has also shown that many firefighters report being physically inactive, despite being aware of the physical nature of their occupation (27–30). In spite of the well-known intense physical requirements of firefighting, many firefighters do not maintain the appropriate levels of physical conditioning that are required for peak performance at work (29, 31, 32). However, studies have shown that firefighters, particularly in the CoCTFRS, that are overworked are predisposed to musculoskeletal injuries and musculoskeletal discomfort (30, 33).

Ageing and obesity predispose firefighters to musculoskeletal injuries (34, 35) and are related to reduced work performance (6, 7, 36). In addition, though firefighters may be relatively healthy, maintaining adequate muscular strength and endurance is essential, as several studies have reported significant relationships between muscular strength and endurance and occupational tasks (6, 7, 36). This may be explained by forceful repetitive movements required by firefighters, such as the forcible entry, hose drag, victim rescue, and heavy equipment carries, that, require high levels of muscular strength and endurance (5–7, 36). Furthermore, it has been consistently reported, in studies performed in different fire departments, globally, that measures of physical fitness, particularly cardiorespiratory fitness and muscular endurance, explained the most variance in occupational performance times in firefighters (3, 5–7, 37, 38). It is apparent that performing firefighting-related tasks with sufficient intensity and efficiency is based on multiple factors mainly associated with a healthy cardiovascular and musculoskeletal system (5, 7, 14, 36, 38). In the CoCTFRS there are no policies or legislations that encourage firefighters to maintain an appropriate level of physical fitness and cardiovascular health to ensure optimal occupational performance, which becomes particularly worrisome given the scarcity of research on the health, wellness, physical fitness, and occupational performance of this population (18, 20).

Previous studies have suggested relationships between physical fitness, cardiovascular health and musculoskeletal health that, collectively, may significantly impact the occupational performance of firefighters (30, 39–42). However, these relationships have not been fully explored, with most studies opting to investigate the relationship between physical fitness and occupational performance, only. This has left a gap in the literature on what the cumulative effect of physical fitness, cardiovascular and musculoskeletal health may have on occupational performance in firefighters, which is particularly relevant for research conducted on firefighters in South Africa. In addition, no study has investigated the determinants of occupational performance, using physical fitness, cardiovascular health and musculoskeletal health in firefighters, in the CoCTFRS. This research will highlight the importance of physical fitness, cardiovascular health, and musculoskeletal health on occupational performance in firefighters, in Africa, where firefighters are understudied. In addition, a better understanding of the parameters that contribute to occupational performance will enable firefighters, instructors and policymakers, particularly in South Africa, to prepare adequately for the physically demanding requirements of the profession. We hypothesise that there will be an inverse relationship between physical fitness and PAT completion times and a positive association between cardiovascular health and musculoskeletal health PAT completion times. A better understanding of the determinants of occupational performance may help support the development of policies standardizing occupational requirements for an acceptable level of physical fitness and cardiovascular health. Therefore, the purpose of this study was to investigate whether physical fitness, cardiovascular health and musculoskeletal health were factors significantly associated with occupational performance in firefighters, in the City of Cape Town Fire and Rescue Service (CoCTFRS).

## 2. Methods

### 2.1. Study design and population

A cross-sectional study design was employed to determine the association between physical fitness parameters (cardiorespiratory fitness, muscular strength and endurance, flexibility and body composition), cardiovascular health (CVD risk factors, CVD risk score, HRV, cardiovascular health index), musculoskeletal health (Musculoskeletal injuries and musculoskeletal discomfort), and occupational performance in a cohort of firefighters. The PAT was administered by the CoCTFRS and was used as the measure of occupational performance in the present study. The study took place between June and August 2022. Written informed consent was obtained from all participants. In total, 1,000 firefighters are currently employed in the CoCTFRS, and using Slovin’s formula, a minimal sample size of 278 firefighters was calculated for this study. Overall, 309 full-time male and female firefighters between the ages of 20 to 65 years from the CoCTFRS were systematically sampled and agreed to participate in the study. Due to the time constraints as a result of the testing, 309 firefighters of the total firefighter population was randomly sampled to participate in the study. However, after the initial health screening, 26 firefighters were excluded due to medical concerns. From the original 309 firefighters, 283 attempted the PAT (92% response rate), and 15 firefighters failed to complete the PAT due to exhaustion. Ethical clearance was granted by the Biomedical Research Ethics Committee (ethical clearance number: BM21/10/9) of the University of the Western Cape. Approval was granted by the Chief Fire Officer, as well as the departments of Research and Policy and Strategy research branch of the City of Cape Town (CCT).

### 2.2. Sampling and participant recruitment

Data collection took place during the annual physical fitness assessment conducted by the CoCTFRS. To ensure consistency of the testing results, a single fire station was used, located in the CCT metropolitan area, to assure the same layout of the PAT, environmental conditions and testing surface. Although the PAT was administered by the fire department, for the present study, all PAT measures were collected and recorded by trained researchers that were familiarised with all the testing instruments and research procedures. Due to time constraints and agreement with the CoCTFRS on the number of firefighters that would be allowed to participate in the study, firefighters were selected using random systematic sampling, where every third firefighter was selected to participate from the 96 platoons (32 fire stations) that participated in this study. Each of the 96 platoons consisted of 8 to 12 firefighters. All full-time firefighters between the age range of 20–65 years were considered. Firefighters excluded were those on administration duty, those on sick leave, those employed as part-time or on a seasonal basis, or those that did not participate in the PAT on the day of testing due to medical concerns or injuries impacting their ability to complete the PAT.

### 2.3. Physical fitness measures

Physical fitness was measured by trained researchers (43) in accordance with the American College of Sports Medicine (ACSM) guidelines (44). Cardiorespiratory capacity was from a validated non-exercise calculation (43) to estimate oxygen consumption (V̇O_2_). For muscular endurance, push-ups and sit-ups tests were used, upper and lower body strength were assessed using the handgrip and leg strength tests and to assess flexibility, the sit-and-reach test was used. Body mass and Lean body mass (LBM) were used as measures of body composition and were assessed using a Tanita^©^ (Tanita^©^, Tokyo, Japan) BC-1000 Plus bioelectrical impedance (BIA) analyser. Cardiorespiratory fitness was estimated using the non-exercise method, applying the following formula: oxygen consumption (V̇O_2max_) = 3.542 + (−0.014 × Age) + (0.015 × Body Mass [kg]) + (−0.011 × Resting Heart Rate) (45). Relative V̇O_2max_ (relV̇O_2max_) was then calculated from absolute V̇O_2max_ (abV̇O_2max_) value generated. For the push-ups and sit-ups tests, firefighters were requested to perform as many repetitions, in a minute, as possible and the test was terminated when firefighters reached volitional fatigue or were unable to maintain a good technique (44). Grip strength was measured using a Takei^®^ 5,401-C handgrip dynamometer and leg strength using a Takei^®^ back and leg strength dynamometer, following standardized protocols and given three attempts with the highest being recorded (44). To ensure accurate results, firefighters were allowed a full recovery between each test. The sit-and-reach required firefighters to reach as forward as far as possible on the ruler of a standardized sit-and-reach box. For a full description of the methods used to assess physical fitness consult the study published by Ras et al. (43).

For relative cardiorespiratory fitness, 42 mL·kg^−1^·min^−1^ (3) was used to indicate the minimum cardiorespiratory fitness needed for firefighting. For measures of absolute cardiorespiratory fitness, grip and leg strength, push-ups and sit-ups and flexibility, the 50th percentile was used to classify firefighters with the minimum required strength, endurance and flexibility measures and categorized as “good.” This percentile was chosen due to the scarcity of objective measures of minimum measures of strength, endurance and flexibility needed for acceptable PAT performance. In total, to calculate the 50th percentile for the fitness measures, 304 firefighters’ data were used. Based on the 50th percentile, firefighters that had an absolute cardiorespiratory fitness of 3.40 L min or above was considered “good.” For muscular strength, a grip strength of 89.9 kg or above and leg strength of 116.5 kg or above were considered “good.” For muscular endurance, a push-ups and sit-ups capacity of 30 repetitions per minute or above were considered to be “good.” For flexibility, a sit-and-reach score of 43 cm or above was considered “good.” Firefighters that fell below the 50th percentile were considered to have a “low” level of muscular strength, muscular endurance and flexibility.

### 2.4. Cardiovascular health measures

In the current study, cardiovascular health was used as an umbrella term and was investigated using several approaches. These approaches included three main subcomponents: CVD risk factors, cardiovascular health metrics and heart rate variability (HRV). Height and waist and hip circumference were assessed using a stadiometer and tape measure, using standardized techniques (44), and using a bioelectrical impedance analysis (BIA) scale body fat percentage (BF%) and weight were measured. CVD risk factors included age, smoking, hypertension, dyslipidaemia, diabetes, obesity and physical inactivity. Cardiovascular health metrics were used to classify firefighters’ cardiovascular health index. The cardiovascular health metrics included an ideal/good body mass index (BMI), blood pressure, non-fasting blood glucose, total cholesterol, level of physical activity, diet and cigarette smoking status. In addition, cardiovascular health index was classified as “good” if firefighters had five to seven metrics rated as ideal, “intermediate” if firefighters had three to four metrics classified as ideal and “poor” if firefighters had zero to two metrics classified as ideal. The 2008 Framingham risk model, developed by D’Agostino et al. (46), was used to assess cardiovascular disease risk of firefighters. Furthermore, the American College of Cardiology (ACC) 10 year atherosclerotic cardiovascular disease (ASCVD) and ASCVD lifetime risk were calculated to assess the cardiovascular disease risk of firefighters (47, 48). For HRV, a Polar^™^ (Polar Electro Oy, Kempele, Finland) H10 heart rate monitor was used, at rest, while firefighters were in a seated position, and analyzed using the Kubio^©^ Software version 3.4.3. Prior to testing, firefighters were asked to remain in a seated position for at least 5 min, thereafter, HRV measures were taken for 5 min. For more information on the methods used to assess cardiovascular health, as well as the classifications of CVD risk factors and cardiovascular health metrics, please refer to the study published by Ras et al. (43).

### 2.5. Classification of musculoskeletal health

Musculoskeletal health was subcategorized as musculoskeletal injuries and musculoskeletal discomfort status, which was further separated into those that sustained an injury while on duty and those that did not, as well as those who were experiencing musculoskeletal discomfort and those who did not. Thereafter, subcategories for those that reported musculoskeletal injuries and musculoskeletal discomfort were categorized based on the location of the musculoskeletal injury or the musculoskeletal discomfort experienced, specifically upper body musculoskeletal injury, lower body musculoskeletal injury, lower back musculoskeletal injury, upper body musculoskeletal discomfort, lower body musculoskeletal discomfort and lower back musculoskeletal discomfort. Musculoskeletal injury and discomfort were measured subjectively via two validated questionnaires, namely the Cornell Musculoskeletal Discomfort Questionnaire (49) and the Nordic Musculoskeletal Questionnaire (50), under the supervision of a trained researcher to ensure the questionnaires were being completed accurately. The Nordic Musculoskeletal Disorders questionnaire comprised 11 questions, divided into three sections and nine categories. This was answered by indicating a “yes” or “no” response to the nine anatomical sites to indicate if a participant did or did not experience injury/trouble to one or more regions during their time as a firefighter. For the Cornell Musculoskeletal Discomfort Questionnaire, the sections were divided into the following twelve body regions: neck, shoulder, upper back, upper arm, low back, forearm and elbow, wrist and hand, hip, thigh, knee, lower leg and foot and ankle. The questionnaire also included data on the frequency of discomfort, the severity and the effect of the discomfort on the ability to do their work.

### 2.6. Occupational performance

#### 2.6.1. Physical ability test

The PAT was used to assess operational performance and was conducted according to the testing protocol of the CoCTFRS. The PAT was developed by the CoCTFRS as part of the fitness and wellness programme in consultation with industry experts. The PAT consists of tasks that are designed to simulate the various duties that firefighters perform, while also attempting the simulate the physical stressors that firefighters are routinely exposed to. To simulate an emergency fire callout, the PAT was conducted while firefighters wore their full PPE equipment and breathing apparatus set. However, firefighters were not required to use the mouthpiece of the breathing apparatus set while performing the PAT to ensure a “full” tank was used for the duration of testing. The PAT consisted of six tasks, which included the step-up, charged hose drag and pull, forcible entry, equipment carry, ladder raise and extension and the rescue drag. Firefighters were required to complete the simulation protocol in under 9 min (540 s) in order to pass. Firefighters passed the PAT if the total completion time was under 540 s. If they failed to complete an individual task, they were, nevertheless, graded competent overall. However, firefighters that failed to pass a specific task were graded “not yet competent” in that task. Firefighters were required to pass the task on the next physical fitness assessment. Firefighters were allowed 20 s of recovery between tasks. The timer was restarted once the recovery period had elapsed, regardless of whether the firefighter was in the starting position. The tasks included:

#### 2.6.2. Step-ups

Firefighters were required to perform 30 step-ups on a 200 mm platform while carrying a high-rise pack weighing 40 kg in total, which consisted of 20 kg weights, strapped together in a twin donut method. The step-up task had a time limit of 90 s to be deemed competent.

#### 2.6.3. Charged hose drag and pull

Firefighters’ were required to place a 45 mm hose line over their shoulder or across the chest and advance the hose tied to a tyre to the 27 meter mark. Thereafter, the firefighters dropped to at least one-knee or in a seated position and pull the hose-line to the 15 meter mark. The firefighters had a time limit of 180 s to complete the test to be deemed competent.

#### 2.6.4. Forcible entry

The forcible entry event required firefighters to pick up a 6 kg sledgehammer and strike a tyre to drive it for a distance of 600 mm. Firefighters were required to complete the task in 60 s to be deemed competent.

#### 2.6.5. Equipment carry

Firefighters were required to remove two foam drums, each weighing 25 kg, from a 1.2 meter-high platform, one at a time, and place them on the ground. The firefighters proceeded to walk both drums, carried in each hand, 25 meters toward and around the first marked position and walk another 25 meters (50 meters in total) back to the starting position. Upon returning, the firefighters placed the foam drums back onto the platform, one at a time. In this task, firefighters were required to complete the task in 60 s to be deemed competent.

#### 2.6.6. Ladder raise and extension

Firefighters were required to walk a seven-to-eight-meter aluminum ladder 6 meters toward the building, raise the ladder using every rung, using the hand-over-hand technique, until stationary against the wall. Immediately thereafter, the firefighters walked to the second pre-position and, using the hauling line, hoisted a 35 kg drum, pulling down the line hand-over-hand, until the fly section reached the pulley and then lower the ladder once again. The firefighters then walked back to the ladder and lowered the ladder using the hand-over-hand technique, returning the ladder it’s the original position. The firefighters were given 90 s to complete this test and deemed competent.

#### 2.6.7. Rescue drag

This event required firefighters to grasp an 80 kg tyre on the shoulders of the harness and drag the tyre 11 meters to a prepositioned mark, perform a 180-degree turn, around the mark, and continue an additional 11 meters toward the finish line. Firefighters were required to complete this task in 60 s to be deemed competent.

### 2.7. Statistical analysis

The data were analysed using SPSS^®^ software, version 28 (Chicago, Illinois, United States). The data were collected, coded and cleaned for errors using the double entry method on Microsoft Excel. Descriptive statistical analyses, such as the median and 25th and 75th percentiles were computed. Mann–Whitney U analysis was performed to determine the difference between PAT completion times based on physical fitness, cardiovascular health and musculoskeletal health groups. Univariable and multivariable linear regressions were performed to determine the independent variables associated with PAT performance as an outcome. Due to the differences in units of measurements for the exploratory and outcome variables, standardized beta coefficients were preferred to interpret the strength of the association. Univariable and multivariable logistic regressions were performed to determine the independent variables associated with PAT pass rates. In the regression analysis, independent (exploratory) variables of physical fitness variables included abV̇O_2max_, relV̇O_2max_, grip strength, leg strength, push-ups, sit-ups, and LBM. Exploratory cardiovascular health variables included age, BMI, BF%, waist circumference, systolic blood pressure, diastolic blood pressure, total cholesterol, non-fasting blood glucose, weekly MET minutes and Framingham risk score. Exploratory variables for musculoskeletal health included musculoskeletal injury, upper body musculoskeletal injury, lower body musculoskeletal injury, lower back musculoskeletal injury, upper body musculoskeletal discomfort, lower body musculoskeletal discomfort and lower body musculoskeletal discomfort. In the multivariable analysis on physical fitness and cardiovascular health parameters, model 2 was adjusted for age and sex and model 3 was adjusted for age, sex, height and weekly METs. Multivariate analysis of covariance (MANCOVA) was conducted to determine the difference/degree of variance between performance categories on the PAT in terms of physical fitness and cardiovascular health. Categories included top performers (75th to 99th percentile), above average performers (50th to 75th percentile), below average performers (25th to 50th percentile), and poor performers (1st to 25th percentile), which was considered as the grouping/independent (fixed factors) variable and physical fitness and cardiovascular health parameters were considered the dependent variables list in the analysis. Covariates adjusted for included age, sex, height and BMI for physical fitness and sex, height and weekly MET minutes for cardiovascular health. Analysis of covariance (ANCOVA) was conducted to determine the difference between performance categories and each dependent variable. Bonferroni correction (0.05/4 = 0.0125) was applied to significant ANCOVA results, and stepwise comparisons were reported. Backward stepwise linear regression models were performed to determine the factors contributing most to PAT completion times. To control for collinearity the VIF and Durbin–Watson statistics were used. A VIF <5 was used to indicate that no substantial collinearity was present and a Durbin–Watson statistic between 1.5 and 2.5 indicated that no autocorrelation was present. For data that were not normally distributed, data were fractionally ranked, and then normalized using the inverse DF, IDF.NORMAL transformation (51). A value of *p* of <0.05 was used to indicate statistical significance.

## 3. Results

In Table 1 we delineate the PAT times according to sex, age-group, cardiovascular health, musculoskeletal health, and physical fitness. The median PAT completion time was 369.5 (293.3, 488.8) seconds. It was higher in women than in men and increased with age (both *p* < 0.001). Firefighters with good relative cardiorespiratory fitness levels, good grip and leg strength, and good push-ups and sit-ups stamina had significantly faster completion times than firefighters with low cardiorespiratory fitness, grip and leg strength and push-ups and sit-ups stamina (all *p* < 0.001). Obese, physically inactive and firefighters with a poor cardiovascular health index had a significantly slower PAT completion time than non-obese physically active and those with an intermediate or good cardiovascular health index (all *p* < 0.001). Firefighters that reported upper body musculoskeletal injury had a slower PAT completion time than those without an injury (*p* = 0.048) and those that reported lower back musculoskeletal injury had a significantly slower completion time (*p* = 0.028).

**Table 1:** Descriptive statistics of firefighters according to age-category, sex, physical fitness, cardiovascular and musculoskeletal health.

Variable	*N*	*X̃* (*p*25^th^–*p*75^th^)
**Demographic characteristics**		
Age (years)	268	36.0 (29.0, 46.0)
Years of experience (years)	268	12.0 (4.0, 19.0)
Height (cm)	268	173.5 (169.1, 178.3)
Weight (kg)	268	81.0 (72.5, 89.9)
**Physical fitness**		
abV̇O_2max_ (L·min^−1^)	268	3.4 (3.3, 3.6)
relV̇O_2max_ (mL·kg^−1^·min^−1^)	268	42.3 (38.4, 46.7)
Grip strength (kg)	268	90.9 (80.0, 101.9)
Leg strength (kg)	268	118.0 (101.6, 135.8)
Push-ups (rpm)	268	30.0 (21.3, 41.0)
Sit-ups (rpm)	268	30.0 (22.0, 36.0)
Flexibility (cm)	268	44.0 (37.0, 50.0)
Lean body mass (kg)	268	61.9 (54.9, 67.7)
**Cardiovascular health**		
Body mass index (kg·m^−2^)	268	26.8 (23.9, 30.0)
Body fat percentage (%)	268	19.5 (14.3, 26.1)
Systolic blood pressure (mmHg)	268	137.2 (124.8, 144.9)
Diastolic blood pressure (mmHg)	268	80.8 (73.7, 90.0)
Total cholesterol (mmol·L^−1^)	268	4.5 (3.9, 5.3)
Low-density lipoprotein cholesterol (mmol·L^−1^)	268	2.6 (2.0, 3.3)
High-density lipoprotein cholesterol (mmol·L^−1^)	268	1.2 (1.1, 2.1)
Triglycerides (mmol·L^−1^)	268	1.4 (0.9, 2.1)
Non-fasting blood glucose (mmol·L^−1^)	268	5.4 (4.9, 6.1)
Framingham risk score (%)	268	0.9 (0.2, 5.5)
Lifetime ASCVD risk score^▲^	266	50.0 (39.0, 50.0)
10-year ASCVD risk score ^¶^	108	5.4 (2.3, 8.9)
**Heart rate variability**		
Heart rate variability (ms)	263	722.0 (633.0, 822.0)
SDNN (ms)	263	33.4 (22.8, 47.5)
RMSSD (ms)	263	24.5 (14.7, 38.9)
LF (Hz)	263	0.09 (0.07, 0.11)
HF (Hz)	263	0.17 (0.16, 0.21)
LF/HF ratio (Hz)	263	2.79 (1.61, 5.20)
**Categories of physical ability test performance (time)**		
Top performers	67	250.0 (226.0, 273.0)
Above average performers	67	330.0 (309.0, 347.0)
Below average performers	67	420.0 (384.0, 441.0)
Poorest performers	67	601.0 (517.0, 728.0)

	*N*	*X̃* (*p*25^th^–*p*75^th^)	*p* ^§^	Pass rate % (*N*)
**Physical ability test (seconds)**				
Total firefighters#	268	369.5 (293.3, 488.8)		81.3 (230)
**Sex**				
Male	239	351.0 (286.0, 441.0)	<0.001**	87.7 (221)
Female	29	654.9 (491.5, 852.5)		29.0 (9)
**Age-group**				
20–29 years	71	337.0 (274.0, 425.0)	<0.001**	92.9 (65)
30–39 years	86	338.0 (272.3, 443.8)		88.0 (81)
40–49 years	67	430.0 (327.0, 550.0)		73.2 (52)
50+ years	43	413.0 (321.0, 594.0)		64.0 (32)
**Physical fitness**				
Good absolute cardiorespiratory fitness	139	322.0 (268.0, 390.0)	<0.001**	93.0 (132)
Low absolute cardiorespiratory fitness	129	441.0 (347.5, 552.0)		69.5 (132)
Good relative cardiorespiratory fitness	139	372.0 (298.0, 488.0)	0.740	83.2 (119)
Low relative cardiorespiratory fitness	129	367.0 (287.5, 492.5)		79.3 (111)
Good grip strength	142	320.5 (261.5, 428.5)	<0.001**	92.4 (134)
Low grip strength	126	423.5 (337.0, 551.0)		68.9 (93)
Good leg strength	143	327.0 (268.0, 409.0)	<0.001**	95.9 (141)
Low leg strength	125	438.0 (337.0, 539.5)		64.7 (86)
Good push-ups stamina	151	327.0 (262.0, 420.0)	<0.001**	90.8 (139)
Low push-ups stamina	117	433.0 (352.5, 559.5)		69.3 (88)
Good sit-ups stamina	151	334.0 (262.0, 438.0)	<0.001**	88.3 (136)
Low sit-ups stamina	117	429.0 (330.0, 535.5)		72.2 (91)
Good flexibility	146	351.5 (286.8, 483.8)	0.321	82.7 (124)
Low flexibility	122	379.5 (304.5, 490.0)		79.2 (103)
**Cardiovascular health**				
Aged	70	428.5 (340.0, 536.3)	<0.001**	69.6 (55)
Young	198	346.5 (277.0, 461.0)		85.8 (175)
Obesity	68	392.0 (324.8, 606.0)	<0.001**	66.7 (50)
Non-obese	200	351.5 (281.0, 460.0)		86.5 (180)
Central obesity	129	390.0 (30.8.0, 535.5)	<0.001**	74.1 (103)
No central obesity	139	337.0 (281.0, 441.0)		88.2 (127)
Hypertension	122	357.0 (292.0, 495.8)	0.930	77.5 (100)
Normotensive	146	380.0 (293.8, 485.0)		84.4 (130)
Dyslipidaemia	86	390.5 (295.5, 532.3)	0.076	77.1 (74)
Normal	182	352.5 (290.5, 455.5)		83.4 (156)
Hypertriglyceridemia	101	377.0 (302.4, 460.5)	0.921	87.2 (95)
Normal	167	354.0 (291.0, 499.0)		77.6 (135)
Diabetes	12	434.0 (325.8, 29.5)	0.237	78.6 (11)
Normal	256	366.0 (293.0, 487.0)		81.4 (219)
Physical inactivity	190	394.5 (312.8, 507.3)	<0.001**	76.8 (146)
Physically active	93	319.5 (255.0, 379.5)		90.3 (84)
Cigarette smoker	97	377.0 (306.5, 460.5)	0.461	85.7 (84)
Non-smoker	171	357.0 (285.2, 495.0)		78.9 (146)
Poor diet	1	725.0 (725.0, 725.0)	0.029*	0.0 (1)
Intermediate diet	44	403.0 (330.0, 529.8)		73.9 (34)
Good diet	223	357.0 (286.0, 477.0)		83.1 (196)
Poor CVHI	83	429.0 (320.0, 533.0)	<0.001**	76.9 (70)
Intermediate CVHI	151	337.0 (270.0, 441.0)		84.4 (135)
Good CVHI	32	393.5 (326.3, 506.0)		78.1 (25)
**Musculoskeletal health**				
Musculoskeletal injury	110	377.5 (297.0, 499.0)	0.150	79.8 (95)
No injury	157	354.0 (290.0, 478.0)		82.2 (134)
Upper body musculoskeletal injury	54	402.0 (317.5, 527.3)	0.048*	76.3 (45)
No injury	214	360.5 (286.0, 465.0)		82.6 (185)
Lower body musculoskeletal injury	63	374.0 (294.0, 490.0)	0.873	81.8 (54)
No injury	205	369.0 (293.0, 488.5)		81.1 (176)
Lower back injury	20	480.5 (328.3, 561.8)	0.028*	68.2 (15)
No injury	248	364.0 (289.5, 477.8)		82.4 (215)
Musculoskeletal discomfort	111	374.0 (281.0, 499.0)	0.722	80.3 (94)
Without musculoskeletal discomfort	157	364.0 (294.0, 483.0)		81.9 (136)
Upper body discomfort	96	373.0 (293.8, 504.3)	0.655	81.9 (81)
Without musculoskeletal discomfort	172	367.5 (293.3, 478.8)		80.2 (149)
Lower body discomfort	64	345.0 (274.0, 482.8)	0.266	80.9 (55)
Without musculoskeletal discomfort	204	370.0 (298.5, 493.8)		81.4 (175)
Lower back discomfort	57	377.0 (298.9, 552.5)	0.161	83.3 (46)
Without musculoskeletal discomfort	211	364.0 (291.0, 478.0)		74.2 (184)

X̃, median; *p*25^th^–*p*75^th^, 25^th^ percentile to 75^th^ percentile; CVHI, cardiovascular health index; ASCVD, atherosclerotic cardiovascular disease; V̇O_2max_, oxygen consumption; L·min^−1^, litres per min; mL·kg^−1^·min^−1^, millilitres per kilogram per minute; mmol·L^−1^, millimole per litre; mmHg, millimetres mercury; kg, kilogram; kg·m^−2^, kilogram per meter squared; cm, centimetres; Hz, hertz; ms, milliseconds; %, percentage; cm, centimetres; rpm, repetitions per minute; SDNN, standard deviation of all normal-to-normal; RMSSD, root-mean-square of successive differences; LF, low-frequency; HF, high frequency; LF/HF, low and high frequency ratio; *p*, significance level; ^§^, Mann–Whitney U analysis; ^¶^, indicates that only firefighters over the age of 40 years were included; ^▴^, indicates that only firefighters under the age of 60 were included.

In Table 2 we describe the linear association between physical fitness and cardiovascular health in relation to PAT completion times. Based on physical fitness, the univariable linear regression analysis indicated that there was a significant negative linear association between abV̇O_2max_, grip strength, leg strength, push-ups, sit-ups, lean body mass LBM and PAT completion times in firefighters. In the multivariable analysis, after adjustment for age and sex, firefighters with a higher abV̇O_2max,_ grip and leg strength, push-ups and sit-ups capacity and LBM performed the PAT significantly faster (all *p* < 0.001). After height and weekly MET minutes were included in the model, firefighters with a higher grip and leg strength, push-ups and sit-ups capacity and LBM performed the PAT significantly faster (all *p* < 0.001). These results support the research hypothesis that occupational performance is inversely associated with physical fitness in firefighters.

**Table 2:** Linear regression assessing the association between physical fitness, cardiovascular health and musculoskeletal health variables, and PAT completion times.

	*B*	Univariable linear models			Multivariable linear models										
		Model 1			Model 2^a^						Model 3^b^				
		SE	*β*	*R* ^2^	Value of *p*	*B*	SE	*β*	*R* ^2^	Value of *p*	*B*	SE	*β*	*R* ^2^	Value of *p*
Model: Physical ability test (s)															
abV̇O_2max_ (L·min^−1^)	−284.41	36.78	−0.182	0.186	<0.001**	−204.04	34.87	−0.308	0.358	<0.001**	−111.43	35.22	−0.159	0.451	0.002**
relV̇O_2max_ (mL·kg^−1^·min^−1^)	−0.206	1.78	−0.007	0.000	0.908	3.368	1.874	0.116	0.282	0.074	−0.47	1.77	−0.016	0.419	0.790
Grip strength (kg)	−5.11	0.53	−0.531	0.265	<0.001**	−3.70	0.564	−0.369	0.377	<0.001**	−2.64	0.55	−0.263	0.476	<0.001**
Leg strength (kg)	−3.12	0.33	−0.509	0.277	<0.001**	−2.09	0.34	−0.341	0.367	<0.001**	−1.57	0.32	−0.257	0.479	<0.001**
Push-ups (rpm)	−5.36	0.71	−0.421	0.175	<0.001**	−4.05	0.72	−0.319	0.356	<0.001**	−4.29	0.63	−0.339	0.520	<0.001**
Sit-ups (rpm)	−6.98	0.96	−0.409	0.165	<0.001**	−5.35	0.93	−0.313	0.355	<0.001**	−5.36	0.82	−0.315	0.511	<0.001**
Sit-and-reach (cm)	−1.48	1.19	−0.076	0.006	0.249	−2.080	1.05	−0.107	0.285	0.049*	−1.76	0.94	0.94	0.427	0.063
Lean body Mass (kg)	−8.28	1.00	−0.453	0.207	<0.001**	−7.55	1.08	−0.413	0.391	<0.001**	−4.25	1.24	−0.233	0.456	<0.001**
Model: Physical ability test (s)															
Age (years)^§^	5.02	1.03	0.286	0.084	<0.001**	5.22	0.92	0.297	0.275	<0.001**	4.81	0.83	0.274	0.430	<0.001**
Body mass index (kg·m^−2^)	5.37	2.45	0.134	0.022	0.029*	−0.32	2.26	−0.008	0.275	0.888	−1.44	2.02	−0.036	0.431	0.477
Bodyfat percentage (%)	6.26	1.09	0.333	0.112	<0.001**	1.69	1.14	0.090	0.281	0.140	0.96	1.03	0.051	0.432	0.352
Waist circumference (cm)	1.13	0.87	0.079	0.007	0.198	0.09	0.89	0.006	0.275	0.920	0.71	0.79	0.050	0.432	0.369
Systolic blood pressure (mmHg)	−1.19	0.71	−0.103	0.008	0.094	−1.10	0.64	−0.09	0.283	0.086	−0.58	0.58	−0.49	0.432	0.319
Diastolic blood pressure (mmHg)	2.02	0.94	0.131	0.024	0.032*	0.69	0.85	0.045	0.277	0.416	0.53	0.76	0.035	0.431	0.483
Total cholesterol (mmol·L^−1^)	8.56	8.41	0.007	0.003	0.310	−6.39	7.64	−0.047	0.277	0.404	−5.70	6.83	−0.42	0.432	0.405
LDL-C (mmol·L^−1^)	9.41	9.98	0.058	0.03	0.347	−6.19	8.91	−0.038	0.276	0.488	−6.26	7.96	−0.038	0.432	0.433
HDL-C (mmol·L^−1^)	−5.97	29.05	−0.013	0.00	0.837	−68.53	25.68	−0.145	0.296	0.008**	−61.74	22.94	−0.130	0.451	0.008**
Triglycerides (mmol·L^−1^)	15.45	9.93	0.095	0.009	0.121	13.32	8.99	0.082	0.281	0.140	7.79	8.06	0048	0.432	0.334
Non-fasting blood glucose (mmol·L^−1^)	0.98	8.18	0.062	0.000	0.905	−4.86	7.13	−0.037	0.278	0.496	−9.08	6.38	−0.068	0.436	0.156
Diet (score)	8.58	5.12	0.102	0.010	0.095	1.82	4.46	0.022	0.275	0.684	5.34	3.99	0.064	0.434	0.183
Weekly MET minutes (MET·min^−1^)^¶^	−0.02	0.00	−0.247	0.061	<0.001**	−0.01	0.00	−0.203	0.313	0.004**	−0.011	0.003	−0.179	0.430	<0.001**
Framingham risk score (%)	5.69	1.99	0.173	0.030	0.005**	5.09	3.00	0.155	0.283	0.099	3.94	2.69	0.120	0.435	0.146
Model: Physical ability test (s)															
Heart rate variability (ms)	−0.24	−0.20	−0.202	0.041	0.001**	−0.23	0.06	−0.190	0.313	<0.001**	−0.135	0.058	−0.112	0.443	0.021*
SDNN (ms)	−2.14	0.49	−0.261	0.065	<0.001**	−1.53	0.45	−0.187	0.300	<0.001**	−0.962	0.413	−0.118	0.438	0.021*
RMSSD (ms)	1.41	0.42	−0.203	0.039	<0.001**	−1.03	0.38	−0.148	0.289	0.006**	−0.600	0.345	−0.087	0.433	0.083
LF (Hz)	−1126.63	412.19	−0.167	0.023	0.007**	−303.40	371.38	−0.045	0.280	0.415	−204.42	332.74	−0.030	0.433	0.540
HF (Hz)	212.42	176.63	0.074	0.005	0.230	83.34	152.09	0.029	0.279	0.584	95.11	136.29	0.033	0.433	0.486
LF/HF (Hz)	−1.00	2.54	−0.024	0.001	0.694	−0.03	2.19	−0.001	0.278	0.989	−0.09	1.97	−0.002	0.432	0.965

*indicates statistically significance <0.05 and **indicates statistical significance <0.01; V̇O_2max_, oxygen consumption; L·min^−1^, litres per min; mL·kg^−1^·min^−1^, millilitres per kilogram per minute; mmol·L^−1^, millimole per litre; mmHg, millimetres mercury; kg, kilogram; kg·m^−2^, kilogram per meter squared; cm, centimetres; Hz, hertz; ms, milliseconds; %, percentage; MET·min^−1^, metabolic equivalent minutes; rpm, repetitions per minute; LDL-C, low-density lipoprotein cholesterol; HDL-C, high-density lipoprotein cholesterol; SDNN, standard deviation of all normal-to-normal; RMSSD, root-mean-square of successive differences; LF, low-frequency; HF, high frequency; LF/HF, low and high frequency ratio; *B*, unstandardized beta coefficient; SE, standard error; *β*, standardized beta coefficient; *R*^2^, *R* squared; §, age was removed as a covariate in the adjustment; ^¶^, weekly MET minutes removed as a covariate in the adjustment.

a Multivariable logistic regression adjusted for age and sex.

b Multivariable logistic regression adjusted for age, sex, height, and weekly MET minutes.

When evaluating cardiovascular health, univariable analysis indicated significant positive associations were found between age, BMI, BF%, diastolic blood pressure, Framingham risk score and PAT completion times, and negative relationship was found between weekly MET minutes and PAT completion times. In the multivariate analysis, after adjustment for sex, an increase in age (*p* < 0.001) was associated with slower PAT completion times. When height and Weekly MET minutes were included, an increase in age (*p* < 0.001) remained associated with slower PAT completion times. For weekly MET minutes, after adjustment for age and sex, an increase in weekly MET minutes (*p* = 0.004) was associated with faster associated PAT completion times. After height was included in the model, firefighters with a higher total weekly MET minutes (*p* < 0.001) remained associated with faster PAT completion times. The results support the hypothesis of the study, however, after robust analysis only age and weekly MET minutes remained significantly associated with PAT completion times.

When evaluating HRV, the univariable analysis indicated that firefighters that had a higher HRV, SDNN, RMSSD and LF performed the PAT significantly faster. After adjustment for age, sex, height and weekly MET minutes, an increase in HRV and SDNN remained associated with faster PAT completion times. These results support the hypothesis that cardiovascular health is positively associated with occupational performance in firefighters.

In Table 3, using logistic regression, we present the association between physical fitness, cardiovascular health and musculoskeletal health variables and PAT performance times in firefighters. Firefighters with good absolute cardiorespiratory fitness, grip strength, leg strength, push-up capacity, and sit-up capacity had increased odds of passing the PAT (all *p* < 0.001). After adjustment for age and sex, a good absolute cardiorespiratory fitness (*p* < 0.001), grip (*p* < 0.012), and leg strength (*p* < 0.001) remained significantly associated to an increased odds of passing the PAT. In model 3, after adjustment for age, sex, height and weekly MET minutes, good absolute cardiorespiratory fitness, grip and leg strength increased the odds of firefighter passing the PAT, which support the hypothesis of the study.

**Table 3:** Logistic regression assessing the association between physical fitness, cardiovascular health, and musculoskeletal health variables and PAT performance (passing the PAT).

	Univariable logistic models		Multivariable logistic models			
	Model 1		Model 2^a^		Model 3^b^	
	OR (95% CI)	Value of *p*	OR (95% CI)	Value of *p*	OR (95% CI)	Value of *p*
Model: PAT performance						
Good absolute cardiorespiratory fitness	5.75 (2.75, 12.00)	<0.001	4.07 (1.72,9.64)	0.001**	3.20 (1.32, 7.75)	0.010*
Good relative cardiorespiratory fitness	1.29 (0.711, 2.36)	0.397	1.42 (0.57, 3.52)	0.453	1.04 (0.40, 2.68)	0.413
Good grip strength	5.78 (2.606, 12.80)	<0.001**	3.16 (1.28, 7.76)	0.012*	2.67 (1.01, 7.06)	0.049*
Good leg strength	12.99 (5.33, 31.68)	<0.001**	5.56 (2.12, 14.57)	<0.001**	4.97 (1.87,13.24)	0.001**
Good push-ups stamina	4.40 (2.26, 8.57)	<0.001**	1.62 (0.73, 3.57)	0.083	2.34 (0.96, 5.71)	0.063
Good sit-ups stamina	2.91 (1.55, 5.44)	<0.001**	0.62 (0.28, 1.37)	0.235	1.69 (0.75, 3.79)	0.206
Good flexibility	1.25 (0.69, 2.28)	0.465	1.54 (0.72, 3.29)	0.264	1.59 (0.73, 3.48)	0.248
Model: PAT performance						
Sex^¶^	0.06 (0.02, 0.14)	<0.001**	0.03 (0.01, 0.08)	<0.001**	0.08 (0.02, 0.26)	<0.001**
Aged^§^	0.38 (0.20, 0.71)	0.002**	0.89 (0.86, 0.93)	<0.001**	0.89 (0.85, 0.93)	<0.001**
Obesity	0.31 (0.17, 0.58)	<0.001**	0.54 (0.86, 0.94)	0.117	0.61 (0.27, 1.38)	0.238
High BF%	0.32 (0.17, 0.60)	<0.001**	0.75 (0.34, 1.67)	0.479	0.91 (0.39, 2.11)	0.832
Central obesity	0.32 (0.17, 0.59)	<0.001**	0.81 (0.36, 1.83)	0.613	0.99 (0.43, 2.29)	0.990
Hypertension	0.64 (0.35, 1.16)	0.140	0.95 (0.43, 2.09)	0.888	0.94 (0.41, 2.26)	0.886
Dyslipidaemia	0.67 (0.36, 1.23)	0.197	1.34 (0.62, 2.90)	0.462	1.25 (0.56, 2.77)	0.588
Hypertriglyceridemia	0.51 (0.26, 0.99)	0.047*	0.47 (0.21, 1.05)	0.065	2.01 (0.89, 4.55)	0.093
Diabetes	0.84 (0.23, 3.11)	0.791	1.47 (0.29, 7.23)	0.636	1.56 (0.30, 8.13)	0.595
Cigarette smoking	1.60 (0.82, 3.12)	0.166	0.91 (0.41, 2.02)	0.813	0.78 (0.33, 1.85)	0.579
Physical inactivity	0.43 (0.22, 0.86)	0.018*	0.34 (0.1.44, 0.82)	0.016*	0.35 (0.13, 0.96)	0.041*
Poor diet	1.54 (0.75, 3.17)	0.244	0.99 (0.41, 2.41)	0.987	1.26 (0.50, 3.17)	0.624
CVHI (Good)						
Intermediate CVHI	1.62 (0.85, 3.09)	0.145	1.56 (0.71, 3.44)	0.268	1.33 (0.58, 3.06)	0.496
Poor CVHI	1.07 (0.41, 1.83)	0.889	1.13 (0.28, 4.63)	0.867	0.88 (0.21, 3.68)	0.861
Model: PAT performance^▲^						
Heart rate variability	1.003 (1.001, 1.005)	0.009**	1.003 (1.001, 1.006)	0.011*	1.003 (1.000, 1.006)	0.039*
SDNN	1.025 (1.010, 1.041)	<0.001**	1.018 (0.999, 1.037)	0.066	1.016 (0.996, 1.037)	0.117
RMSSD	1.018 (1.006, 1.031)	0.005**	1.015 (0.999, 1.032)	0.060	1.014 (0.997, 1.032)	0.104
LF	34.7222 (4.100, 294.071)	0.001**	3.225 (0.227, 45.790)	0.387	4.850 (0.298, 79.049)	0.267
HF	0.222 (0.002, 29.422)	0.546	0.216 (0.002, 28.821)	0.540	1.281 (0.003, 537.546)	0.936
LF/HF	1.013 (0.945, 1.087)	0.709	0.898 (0.902, 1.085)	0.989	0.977 (0.887, 1.076)	0.642
Model: PAT performance						
Musculoskeletal injury	0.86 (0.47, 1.59)	0.634	1.66 (0.77, 3.56)	0.193	1.63 (0.74, 3.58)	0.228
Upper limb injuries	0.68 (0.34, 1.35)	0.270	0.0.83 (0.35, 1.95)	0.665	0.86 (0.35, 2.12)	0.748
Lower limb injuries	0.95 (0.47, 1.94)	0.897	0.46 (0.18, 1.15)	0.095	0.47 (0.18, 1.19)	0.110
Lower back injury	0.46 (0.18, 1.19)	0.111	0.39 (0.13, 1.22)	0.106	0.39 (0.12, 1.27)	0.118
Musculoskeletal discomfort	0.90 (0.49, 1.65)	0.736	1.16 (0.55, 2.45)	0.699	1.09 (0.50, 2.6)	0.833
UBMSD	0.89 (0.48, 1.66)	0.730	0.95 (0.44, 2.05)	0.904	0.90 (0.49, 1.68)	0.746
LBMSD	0.97 (0.48, 1.94)	0.925	1.32 (0.56, 3.10)	0.519	0.97 (0.49, 1.95)	0.938
Lower back discomfort	0.58 (0.29, 1.14)	0.112	0.59 (0.26, 1.39)	0.232	0.65 (0.27, 1.57)	0.339

*indicates statistically significance <0.05 and **indicates statistical significance <0.01. OR, odds ratio; SE, standard error; CI, confidence intervals; ^¶^, sex removed as a covariate in the adjustment; ^§^, age was removed as a covariate in the adjustment; ^▴^, continuous independent variable; SDNN, standard deviation of all normal-to-normal; RMSSD, root-mean-square of successive differences; LF, low-frequency; HF, high frequency; LF/HF, low and high frequency ratio.

a Multivariable logistic regression adjusted for age and sex.

b Multivariable logistic regression adjusted for age, sex, height, and weekly MET minutes.

Univariable analysis found that age, obesity (*p* = 0.002), high BF% (*p* < 0.001), central obesity (*p* < 0.001), hypertriglyceridemia (*p* = 0.047), and physical inactivity (*p* = 0.018) decreased the odds of firefighters passing the PAT. After adjustment for age, height and weekly MET minutes female (*p* < 0.001) firefighters were less likely to pass PAT. When adjusting for sex, height and weekly MET minutes, aged firefighters were less likely to pass the PAT (*p* < 0.001). After adjustment for age, sex, height and weekly MET minutes physically inactive (*p* = 0.018) firefighters were less likely to pass the PAT. Moreover, we found that an increase in HRV, SDNN, RMSSD and LF were significantly associated with an increase in PAT pass rates (all *p* < 0.01). After adjustment, only an increase in HRV was associated with an increased odds of firefighters passing the PAT (*p* = 0.039).

A backward stepwise multiple regression reported that the variation of abV̇O_2max_, grip strength, leg strength, push-ups, sit-ups and LBM used in the model explained a significant proportion (49.0%) of the variation observed in PAT completion times [*F*(7, 256) = 40.1, *p* < 0.001] (Supplementary Table S1). The variation in age, BMI, systolic blood pressure, diastolic blood pressure and weekly MET minutes used in the model explained a significant proportion (30.1%) of the variation of PAT completion times [*F*(6, 256) = 18.4, *p* < 0.001]. The model that included both physical fitness and cardiovascular health parameters showed that the variation in weekly MET minutes, BF%, abV̇O_2max_, grip strength, leg strength and sit-ups explained the highest proportion (50.5%) on the variation of PAT completion times [*F*(6, 256) = 45.6, *p* < 0.001]. These results are consistent with the hypothesis, indicating that physical fitness and cardiovascular health contribute significantly to the occupational performance in firefighters.

In Table 4 we explore the differences between the physical fitness and cardiovascular health of firefighters based on performance on the PAT. The results of the MANCOVA indicated that there was a significant difference between the performance categories on the PAT and physical fitness, where firefighters with higher levels of physical fitness were more likely to be better performers on the PAT, controlling for age, sex, height and BMI [*F*(9, 750) = 3.5, *p* < 0.001, Pillai’s Trace *V* = 0.305]. ANCOVA indicated that abV̇O_2max_ (*p* < 0.001), relV̇O_2max_ (*p* = 0.032), grip strength (*p* = 0.001), leg strength (*p* < 0.001), push-ups (*p* < 0.001), sit-ups (*p* < 0.001), and LBM (*p* < 0.001) was significantly different and more likely to be higher between highest and lowest performance groups on the PAT. After Bonferroni correction, abV̇O_2max_, grip strength, leg strength, push-ups and sit-ups remained robust to the adjustment. Notably, relV̇O_2max_ and LBM were no longer significant after the correction.

**Table 4:** Multivariable comparisons evaluating the difference between physical fitness and cardiovascular health parameters based on physical ability test performance.

Variable	*V*	G1	G2	G3	G4	*F*	Value of *p*						
								Stepwise comparisons^†^					
		Top performers (*n* = 65)	Above average (*n* = 67)	Below average (*n* = 67)	Poorest performers (*n* = 65)		Overall	G1 vs. G2	G1 vs. G3	G1 vs. G4	G2 vs. G3	G2 vs. G4	G3 vs. G4
		x̄ (SE)	x̄ (SE)	x̄ (SE)	x̄ (SE)								
Pillai’s Trace	0.305					3.5	<0.001**						
abV̇O_2max_ (L·min^−1^)		3.59 (0.02)	3.46 (0.02)	3.39 (0.02)	3.36 (0.02)	6.8	<0.001**	0.789	0.002**	<0.001**	0.121	0.015*	1.000
relV̇O_2max_ (mL·kg^−1^·min^−1^)		43.20 (0.31)	43.24 (0.28)	42.40 (0.28)	42.09 (0.32)	2.9	0.032*	1.000	0.383	0.149	0.223	0.065	1.000
Grip strength (kg)		97.11 (1.87)	90.44 (1.68)	88.23 (1.69)	86.11 (1.94)	5.6	0.001**	0.038*	0.004**	0.001**	1.000	0.638	1.000
Leg strength (kg)		128.2 (3.18)	120.84 (2.87)	113.79 (1.89)	107.67 (3.31)	6.3	<0.001**	0.455	0.007**	<0.001**	0.512	0.025*	0.971
Push-ups (rpm)		39.43 (1.54)	34.29 (1.38)	28.93 (1.39)	24.61 (1.59)	14.2	<0.001**	0.064	<0.001**	<0.001**	0.042*	<0.001**	0.247
Sit-ups (rpm)		34.72 (1.17)	29.58 (1.05)	28.68 (1.06)	23.55 (1.21)	12.4	<0.001**	0.005**	0.001**	<0.001**	1.000	0.002**	0.009**
Sit-and-reach (cm)		45.92 (1.17)	43.02 (1.06)	42.14 (1.06)	41.82 (1.22)	2.3	0.082	–	–	–	–	–	–
Lean body Mass (kg)		61.75 (0.68)	61.24 (0.61)	61.00 (0.62)	58.76 (0.71)	3.1	0.027*	1.000	1.000	0.033*	1.000	0.067	0.098
Pillai’s Trace	0.393					2.7	<0.001**						
Age (years)		33.45 (1.27)	36.31 (1.18)	38.62 (1.19)	44.39 (1.31)	11.3	<0.001**	0.562	0.024*	<0.001**	1.000	<0.001**	0.008**
BMI (kg·m^−2^)		27.33 (0.58)	27.27 (0.54)	27.14 (0.55)	27.33 (0.60)	0.0	0.994	–	–	–	–	–	–
WC (cm)		90.29 (1.59)	92.02 (1.49)	93.33 (1.51)	96.47 (1.65)	2.2	0.084	–	–	–	–	–	–
BF% (%)		20.94 (1.12)	20.13 (1.04)	20.33 (1.06)	23.29 (1.15)	1.6	0.197	–	–	–	–	–	–
SBP (mmHg)		136.52 (1.97)	137.40 (1.83)	132.82 (1.86)	136.83 (2.03)	1.3	0.291	–	–	–	–	–	–
DBP (mmHg)		79.47 (1.52)	83.44 (1.41)	81.27 (1.44)	82.98 (1.57)	1.5	0.222	–	–	–	–	–	–
NFBG (mmol·L^−1^)		5.89 (0.18)	5.29 (0.16)	5.67 (0.18)	5.89 (0.18)	2.9	0.031*	0.064	1.000	1.000	0.657	0.098	1.000
TC (mmol·L^−1^)		4.64 (0.17)	4.70 (0.16)	4.69 (0.16)	4.92 (0.18)	0.4	0.718	–	–	–	–	–	–
LDL-C (mmol·L^−1^)		2.70 (0.14)	2.67 (0.14)	2.64 (0.13)	2.91 (0.15)	0.7	0.565	–	–	–	–	–	–
HDL-C (mmol·L^−1^)		1.39 (0.05)	1.29 (0.04)	1.23 (0.04)	1.26 (0.05)	2.1	0.091	–	–	–	–	–	–
Triglycerides (mmol·L^−1^)		1.36 (1.14)	1.55 (0.13)	1.91 (1.33)	1.81 (1.15)	2.9	0.033*	0.188	0.198	0.216	0.187	0.201	0.197
Diet (score)		9.46 (0.28)	9.92 (0.26)	9.93 (0.26)	10.48 (0.29)	1.9	0.127	–	–	–	–	–	–
Weekly METs (MET·min^−1^)		3983.5 (369.4)	4139.4 (343.8)	2716.5 (349.8)	1800.7 (381.7)	7.9	<0.001**	1.000	0.091	<0.001**	0.024*	<0.001**	0.464
Framingham risk score (%)		1.03 (0.66)	2.56 (0.62)	4.04 (0.63)	6.09 (0.68)	8.9	<0.001**	0.506	0.008**	<0.001**	0.564	0.001**	0.162

Univariable comparisons of each parameter of physical fitness and cardiovascular health. Stepwise comparisons of each performance category after Bonferroni correction. *indicates statistical significance <0.05 and **indicates statistical significance <0.01. Physical fitness MANCOVA: adjusted for age, sex, height, and body mass index. Cardiovascular health MANCOVA: adjusted for sex, height and weekly MET minutes. x̄, adjusted mean; SE, standard error; ^†^, indicates Bonferroni correction; *V*, Pillai’s trace; *F*, test statistic ANOVA; G1, top performers; G2, above average performers; G3, below average performers; G4, below average performers; V̇O_2max_, oxygen consumption; L·min^−1^, litres per min; mL·kg^−1^·min^−1^, millilitres per kilogram per minute; mmol·L^−1^, millimole per litre; mmHg, millimetres mercury; kg, kilogram; kg·m^−2^, kilogram per meter squared; cm, centimetres; %, percentage; MET·min^−1^, metabolic equivalent minutes; rpm, repetitions per minute.

Based on cardiovascular health, MANCOVA indicated that there was a significant difference between the performance categories on the PAT and cardiovascular health parameters, where firefighters with worse a cardiovascular health were more likely to be poorer performers on the PAT, controlling for sex, height and weekly MET minutes [*F*(14, 741) = 2.7, *p* < 0.001, Pillai’s Trace *V* = 0.393]. ANCOVA indicated that age (*p* < 0.001), non-fasting blood glucose (*p* = 0.031), triglycerides (*p* = 0.033), weekly MET minutes (*p* < 0.001), and Framingham risk score (*p* < 0.001) was significantly different and more likely to be lower between the highest and lowest performance groups. After Bonferroni correction age, weekly MET minutes and Framingham risk score remained robust to the correction. Non-fasting blood glucose and triglycerides did not remain significant after Bonferroni correction.

## 4. Discussion

Our results indicated that younger, non-obese firefighters, with a higher physical fitness and lower cardiovascular disease risk score, had significantly faster PAT completion times (better occupational performance) and higher pass rates. In addition, the top performing (highest quartile) firefighters had significantly higher abV̇O_2max_, grip and leg strength and push-ups and sit-ups capacity compared to the poorest performers and, unsurprisingly, firefighters with a higher physical fitness level performed best on the PAT, while having the highest pass rates. The results were consistent with what has been reported in the literature, which consistently shows that firefighters that have a higher cardiorespiratory fitness level, muscular strength and endurance, and a more favourable body composition perform best on the occupational performance simulation protocols (5, 13, 14, 52). Physically fit firefighters are likely able to sustain a high work-rate for the duration of the occupational testing, completing the sequence of tasks faster. We found that firefighters aged 45 years or older, with a BMI of 30 kg·m^−2^ or higher had the slowest times on the PAT, especially those with other comorbidities. These results are consistent with previous studies, which indicated that age and obesity are significant predictors of occupational performance in firefighters (5, 13, 53–55). This is likely due to the age-related decline in cardiorespiratory fitness, muscular strength and lean body mass, that is often accompanied by the accumulation of fat mass (32, 56–58). The association between a better overall cardiovascular health status and better occupational performance may be indirectly related to these participants, generally, being more physically active and having a better physical fitness level, particularly cardiorespiratory fitness, which has been shown to be significantly associated in the literature (29, 59, 60). The results may inform policy makers and fire department heads in Cape town on the importance of firefighters maintaining an acceptable level of physical fitness, cardiovascular health and musculoskeletal health in order to perform their duties with sufficient rigor and efficiency, which will also contribute toward their overall health and wellbeing while in the fire service. These results also highlight the importance of annual health screenings and physical fitness testing for firefighters.

The current results indicated that higher absolute cardiorespiratory fitness was significantly related to faster occupational completion times in firefighters. Firefighters who had the highest estimated cardiorespiratory fitness levels, were the top performers. This is supported by previous literature which reported that measured cardiorespiratory fitness is essential for occupational performance in firefighters (13, 61, 62). After adjustment for covariates, the differences in absolute and relative cardiorespiratory fitness were small, perhaps due to cardiorespiratory fitness being estimated in the present study, rather being measured using physical testing. Previous studies (3, 7, 63) have recommended that firefighters should maintain a cardiorespiratory standard of 42 mL·kg^−1^·min^−1^. However, in the present study, meeting the minimum cardiorespiratory fitness standard of 42 mL·kg^−1^·min^−1^ was not a significant factor in passing the PAT, rather absolute cardiorespiratory fitness played a more central role in PAT performance. Siddall et al. (52, 63) noted that in firefighters in the United Kingdom, the required oxygen consumption (and thus the percent of V̇O_2max_ needed) fluctuates significantly based on the task performed, which may explain why this standard was not related to passing the current PAT. Although meeting the standard of 42 mL·kg^−1^·min^−1^ was not needed to pass the PAT in the current study, firefighters with a higher absolute and relative V̇O_2max_ completed the PAT significantly quicker. In the current results, after adjustment, the top performers and poorest performers showed a mean absolute cardiorespiratory fitness level of 3.59 vs. 3.36 L·min^−1^ and 43.2 vs. 42.1 mL·kg^−1^·min^−1^, and a median completion time of 250.0 s vs. 601.0 s. Fitter firefighters may be able to sustain a high physical work rate for an extended period of time (52, 64), allowing firefighters to complete the tasks swiftly. Firefighters were allowed a 20 s full recovery period between tasks, which may account for the absence of significance between firefighters that met the requirement of 42 mL·kg^−1^·min^−1^ and those that did not. A similar observation was made by Rhea et al. (13) which noted that providing firefighters a full recovery period between tasks lessened the cardiovascular fitness level required for each task. However, the study used a 10 min recovery, which is significantly higher than the present study.

We found that an increase in grip and leg strength were significantly associated to faster PAT completion times. Similarly, Rhea et al. (13) reported that in a cohort of 20 full-time firefighters from the Unites States, bench press (*r* = −0.66), squat (*r* = −0.30), and grip strength (*r* = −0.71) were inversely related to occupational performance. Moreover, higher strength levels in either the upper or lower body enhanced the performance on specific tasks that taxed either the upper body or lower body more. In addition, firefighters who performed best on tasks, such as the hose drag and pull, which equally taxed the upper and lower body, were those that had the highest upper and lower body strength levels (13). This was supported by studies conducted by Michaelides et al. (5, 6) that reported that in a cohort of 72 firefighters from Arkansas, United States, abdominal (*r* = −0.53), bench press (*r* = −0.31), and squat (*r* = −0.22) strength were inversely related to ability test performance, performing the ability test quicker than weaker firefighters. Chizewski et al. (7) corroborates these findings where the study noted that in 89 full-time from Illinois, United States, firefighters an increase in bench press strength significantly reduced the total occupational performance completion time and for each task in the battery. A study noted that between the fastest and slowest performers there was a 13% difference in strength levels (3). von Heimburg et al. (38) reported that in firefighters from Trondheim, Norway, a minimum muscular strength level is required to perform tasks efficiently and beyond this point little benefit is gained for an increase in muscular strength capacity. The PAT test used in the present study required firefighters to have the ability to produce substantial force, particularly in the step-up and hose drag and pull tasks, which is designed to exhaust the lower extremities. This is not exclusive to the current study, as previous studies have noted that leg strength and grip strength have been related to the stair climb and hose drag tasks, respectively (13, 65–67). Notably, in the current study, an increase in leg strength was associated with an increase in the likelihood (OR = 4.97) of passing the PAT, more so than the other physical fitness variables. In contrast, though studies have found leg strength was significantly associated with occupational performance, these studies did not report similar strengths in the association between leg strength and occupational performance, as in the present study (5, 6, 13, 14). It is likely that different occupational performance testing protocols tax different aspects of physical fitness and put emphasis on different muscle groups, making them very particular to the fire departments testing protocols (7, 36, 38).

We noted that sit-ups and push-ups were inversely associated to PAT completion times. Michaelides et al. (6) reported that sit-ups (*r* = −0.27) and push-ups (*r* = −0.31) were inversely related to occupational performance in firefighters. Another study by Michaelides et al. (5) reported that muscular endurance was significant in the prediction model for occupational performance in firefighters. Similarly, Chizewski et al. (7) reported that sit-ups (*r* = −0.407) and push-ups (*r* = −0.380) were inversely related to occupational performance and, together, explained 45% of the variance in occupational performance times when entered into the regression model. Rhea et al. (13) further supports this where the study reported that higher endurance capacity in the row (*r* = −0.61), bench press (*r* = −0.71), shoulder press (*r* = −0.73), biceps curl (*r* = −0.69), squat (*r* = −0.47), abdominal curl (*r* = −0.24), and handgrip (*r* = −0.25) tests were inversely related to occupational performance times. This was also supported by Williford et al. (36) who found that in a cohort of firefighters from Alabama, United States, push-ups (*r* = −0.38) and sit-ups (*r* = −0.32) were negatively correlated with occupational performance in firefighters. However, did not contribute significantly to the prediction model. The PAT, and other occupational performance tests, require firefighters to sustain a minimum amount of muscular force for a number of repetitions (12). Inherently, firefighters with high levels of muscular stamina would perform better without experiencing substantial levels of fatigue. The results of the present study indicated that flexibility was not significantly associated with occupational performance, which is consistent to previous literature (6, 7, 14). In contrast, Michaelides et al. (5) noted that higher flexibility, using the sit-and-reach test, was not significantly associated with occupational performance, however, flexibility added significantly to their prediction model. Williford et al. (36) reported that higher flexibility did not improve overall occupational performance but improved the performance on the stair climb (*r* = −0.25). Although flexibility may not consistently be significantly related to occupational performance, good flexibility may be important for reducing the incidence of injuries in firefighters (68), and, possibly, in the performance of certain tasks, such as the stair climb (12, 36).

We found that age, obesity, Framingham risk score and physical activity levels of firefighters were significantly associated with PAT completion times and PAT pass rates. Michaelides et al. (6) noted that age was negatively related (*r* = −0.42) to ability test completion times. Similarly, Myhre et al. (61) reported that, in 222 full-time firefighters from the United States consisting of one army and seven air force base fire departments, age (*r* = 0.38) was positively related to occupational performance. Studies by Chizewski et al. (7), skinner et al. (14), and Williford et al. (36) reported that, although age was not related to total completion times, aging was positively related to the self-contained breathing apparatus (SCBA) crawl (*r* = 0.359), the dummy drag (*r* = 0.389), and the stair climb (*r* = 0.48) in each study, respectively. Michaelides et al. (6) reported that BMI (*r* = 0.34) and BF% (*r* = 0.57) was positively related to completion times in firefighters. Another study by Michaelides et al. (5) reported that BF% (*r* = 0.41) was positively correlated with occupational performance completion times. Similarly, Schonfeld et al. (62) reported that in a cohort of 20 full-time firefighters from the Kennedy Space centre Florida, United States, BF% (0.467) was moderately related to occupational performance times in firefighters. This was supported by Williford et al. (36) who reported that BF% (*r* = 0.30) was positively related to total occupational performance completion times and related to the completion of all tasks, except the hose advance. Skinner et al. (14) noted that in a cohort of 42 Australian full-time firefighters, BMI was not related to occupational performance, however, BF% was positively related to completion times (*r* = 0.481). Chizewski et al. (7) reported that BMI was not related to total occupational performance completion times, however, BMI was negatively related to the performance of specific tasks, such as the SCBA crawl (*r* = −0.276), and negatively related to the hose advance (*r* = −0.272), and ladder raise (*r* = −0.274). The combination of the general attrition in physical fitness and accumulation of fat mass that is associated with aging (32, 56–58), increases the non-functional weight firefighters are required to overcome (12), providing a possible explanation for the reduction in their occupational performance. Higher levels of cardiorespiratory fitness and weekly physical activity has been linked to a favourable cardiovascular health profile and cardiovascular functioning (69, 70), which may directly relate to better occupational performance in firefighters (12, 64, 71).

The current study found that HRV, SDNN, RMSSD and LF were significantly and inversely associated with occupational completion times in firefighters. This was supported in a study by Lesniak et al. (72) who reported that HRV was correlated with occupational performance in firefighters. Porto et al. (73) noted that SDNN and RMSSD were higher and LF was dominant in fitter firefighters. Similarly, a systematic review conducted by Tomes et al. (74) noted that HRV was a reliable indicator of key physical fitness and occupational performance parameters (74). The results of the current study and previous research suggests that firefighters with higher parasympathetic dominance, who are in a more relaxed state, may perform better on occupational performance tasks (74, 75). This measure may be an important indicator for firefighters’ overall cardiovascular health and fitness levels, particularly in relation to firefighters’ work performance (72, 74, 76).

We found that weekly MET minutes, BF%, abV̇O_2max_, grip strength, leg strength and sit-ups explained 50.5% of the variance in PAT completion times in firefighters. Similarly, Michaelides et al. (5) noted that the fitness parameters, which included flexibility, sit-ups, push-ups, BF%, 1-RM bench press and squat explained 59% of the variance in occupational performance completion times in firefighters. Davis et al. (37) supported this and noted that push-ups, sit-ups, and grip strength explained 54% of the variability in occupational performance completion times in firefighters. In addition, Davis et al. noted that BF%, LBM and cardiorespiratory fitness were significantly associated with fatigue resistance in firefighters. Furthermore, the study found that aging, LBM and grip strength explained 60.6% of the variance in PAT pass rates. Williford et al. (36) reported that run time, pull-ups and fat free weight explained 53% of the variance in occupational performance completion times. In contrast, Siddal et al. (52) noted that age and LBM did not contribute to the strength of the regression models and that abV̇O_2max,_ fat mass/BF% accounted for the most variance in occupational performance with 56.7 and 57.2%, respectively. Williams-Bell et al. (77) reported that abV̇O_2max_ or relV̇O_2max_, body mass and handgrip strength were significantly associated with occupational performance in firefighters and accounted for 65–71% of the variance in occupational performance completion times. Furthermore, the final model removed push-ups from the equation, which also seen in the final model in the current study (77). Moreover, in the current study, grip and leg strength remained significant in the regression model. In contrast, Williams-Bell et al. (77) reported that measures of strength and power were no longer significant predictors after absolute cardiorespiratory fitness was included. However, the study indicated that when relative cardiorespiratory fitness was used as the only measure of cardiorespiratory fitness, grip strength was significantly related to occupational performance. The result of the backward stepwise multivariable regression suggests that fitter and stronger firefighters, with a higher muscular endurance and favourable body composition perform best on the PAT and are more likely to pass the occupational performance test.

The current results found that musculoskeletal health was not a significant contributing factor in the model to predict firefighters’ performance on the PAT. This was supported by MacDermid et al. (78), where the study noted no relationship between task performance and self-reported work limitations in firefighters from Ontario, Canada. The study noted that those who reported lower limb discomfort took 10 s longer to complete the stair climb task. It is intuitive to anticipate that that there would be an association between occupational performance and musculoskeletal health in firefighters. The failure to find a significance may indicate that firefighters were completely recovered from any injury, or the discomfort may not have been significant enough to cause a decrease in absolute performance. Perhaps, having discomfort in specific regions may limit performance of tasks that tax that specific region that firefighters are experiencing discomfort in. However, firefighters may make up for this by performing well in the other tasks, which may have been the case in the present study.

This is the first study of its nature to be conducted in this population. Therefore, the results of the present study may contribute meaningfully toward informing policy makers on the need for the development of new policies and legislation aimed at encouraging firefighters to either maintain or improve their levels of physical fitness, cardiovascular health and musculoskeletal health in the CoCTFRS. The absence of research on firefighters in Africa, and particularly in South Africa, presumably contributed to the stagnated development of new policies focused on the occupational health and wellbeing of firefighters (18). This arguably has led to the progressive deterioration in firefighter health and wellness that has become problematic in firefighters in South Africa (18, 30). This research highlights the need for annual health screening and physical fitness testing of firefighters and emphasizes the value that routine testing may provide for the fire and rescue service in Cape Town. Firefighters have reported that two primary barriers to physical activity were a lack of resources, such as facilities and equipment to exercise regularly, and the lack of energy to exercise while on- or off-duty (79). While many firefighters reported that they opted for unhealthy snacks, because of the unpredictable nature of emergency callouts and the need for quick meals (21, 22). Policy makers and fire department heads in Cape Town should take this into consideration when implementing policies to ensure that firefighters remain sufficiently active in order to either maintain or improve their physical fitness. While also ensuring that they are educated on the benefits of opting for healthier diets while on- or off-duty. In addition, implementing minimum requirements for cardiorespiratory fitness, and muscular strength and endurance are necessary to ensure that firefighters are physically capable of performing their duties (33, 80). This will also help maintain good CVH in firefighters to ensure that they are not at risk for CVD-related events while on duty (4, 81).

### 4.1. Strengths and limitations

This was the first study investigating the association between physical fitness, cardiovascular health, musculoskeletal health and occupational performance in Africa. The measures for physical fitness, cardiovascular health and occupational performance were objectively measured using standardized and validated instruments (43). Furthermore, this paper adds novel information into an area which has been understudied in firefighters, particularly in a South African context. There are, however, several limitations of the present study. Firstly, this study used a cross-sectional study design, which precludes the inference of causal relationships. Secondly, musculoskeletal injuries and musculoskeletal discomforts were self-reported, which may have introduced reporting bias. Thirdly, cardiorespiratory fitness was estimated using a non-exercise calculation (and we found little variability in relative V̇O_2max_ among groups), which may have reduced the expected associations between relative cardiorespiratory fitness and other variables. Lastly, female firefighters were under-represented, limiting the generalizability of our findings to the female firefighter population.

## 5. Conclusion

The present study provides evidence that multiple parameters of physical fitness and cardiovascular health are related to overall occupational performance in firefighters. The findings show that younger, leaner, fitter and stronger firefighters with a favourable cardiovascular health profile performed significantly better and were most likely able to pass each individual task. The results emphasize the need for firefighters to maintain high levels of physical fitness and a good cardiovascular health profile to ensure they maintain an acceptable level of occupational performance. This study adds novel research into the field, highlighting the factors that contribute significantly to occupational performance in firefighters, particularly in a South African context, where firefighters are understudied. The results of this study may be used by municipal fire departments to highlight the need for developing physical fitness standards to ensure the cardiovascular and musculoskeletal health of firefighters, to improve the career longevity and occupational performance of firefighters. By implementing regular physical activity programmes and promoting minimum fitness standards, fire departments could improve the services provided by firefighters, protect firefighters’ health, reduce the likelihood of civilian casualties and secure essential infrastructure.

## Data availability statement

The original contributions presented in the study are included in the article/Supplementary material, further inquiries can be directed to the corresponding author.

## Ethics statement

The studies involving humans were approved by the Biomedical Research Ethics Committee (ethical clearance number: BM21/10/9) of the University of the Western Cape (UWC). The studies were conducted in accordance with the local legislation and institutional requirements. The participants provided their written informed consent to participate in this study. Written informed consent was obtained from the individual(s) for the publication of any potentially identifiable images or data included in this article.

## Author contributions

JR, DS, ES, AK, and LL contributed to the conception and design of the study, proofread, and edited the drafts of the manuscript. JR organized the database, performed the statistical analysis, collected the data, and wrote the first draft of the manuscript. All authors contributed to the article and approved the submitted version.

## Funding

This research was funded by the National Research Foundation (NRF) (grant number 141282) and The Ryoichi Sasakawa Young Leaders Fellowship Fund (SLYFF). Neither funding bodies were involved in the study design, data collection or interpretation of the data.

## Conflict of interest

The authors declare that the research was conducted in the absence of any commercial or financial relationships that could be construed as a potential conflict of interest.

## Publisher’s note

All claims expressed in this article are solely those of the authors and do not necessarily represent those of their affiliated organizations, or those of the publisher, the editors and the reviewers. Any product that may be evaluated in this article, or claim that may be made by its manufacturer, is not guaranteed or endorsed by the publisher.

## References

[1] Smith DL Haller JM Korre M Sampani K Porto LGG Fehling PC. The relation of emergency duties to cardiac death among US firefighters. Am Chem J. (2019) 123:736–41. doi: 10.1016/j.amjcard.2018.11.049, PMID: 30567633

[2] Le AB Smith TD McNulty LA Dyal MA Dejoy DM. Firefighter overexertion: a continuing problem found in an analysis of non-fatal injury among career firefighters. Int J Environ Res Public Health. (2020) 17:7906. doi: 10.3390/ijerph17217906 33126593 PMC7663299

[3] von Heimburg ED Rasmussen AKR Medbø JI. Physiological responses of firefighters and performance predictors during a simulated rescue of hospital patients. Ergonomics. (2006) 49:111–26. doi: 10.1080/00140130500435793, PMID: 16484140

[4] Smith DL DeBlois JP Kales SN Horn GP. Cardiovascular strain of firefighting and the risk of sudden cardiac events. Exerc Sport Sci Rev. (2016) 44:90–7. doi: 10.1249/JES.0000000000000081, PMID: 27111479

[5] Michaelides MA Parpa KM Thompson J Brown B. Predicting performance on a firefghter’s ability test from fitness parameters. Res Q Exerc Sport. (2008) 79:468–75. doi: 10.1080/02701367.2008.10599513, PMID: 19177948

[6] Michaelides MA Parpa KM Henry LJ Thompson GB Brown BS. Assessment of physical fitness aspects and their relationship to firefighters’ job abilities. The. J Strength Cond Res. (2011) 25:956. doi: 10.1519/JSC.0b013e3181cc23ea, PMID: 20703167

[7] Chizewski A Box A Kesler R Petruzzello SJ. Fitness fights fires: exploring the relationship between physical fitness and firefighter ability. Int J Environ Res Public Health. (2021) 18:11733. doi: 10.3390/ijerph182211733, PMID: 34831490 PMC8625752

[8] Sothmann MS Gebhardt DL Baker TA Kastello GM Sheppard VA. Performance requirements of physically strenuous occupations: validating minimum standards for muscular strength and endurance. Ergonomics. (2004) 47:864–75. doi: 10.1080/00140130410001670372, PMID: 15204279

[9] Sothmann MS Saupe KW Jasenof D Blaney J Fuhrman SD Woulfe T. Advancing age and the cardiorespiratory stress of fire suppression: determining a minimum standard for aerobic fitness. Hum Perform. (1990) 3:217–36. doi: 10.1207/s15327043hup0304_1

[10] Swank AM Adams KJ Barnard KL Berning JM Stamford BA. Age-related aerobic power in volunteer firefighters, a comparative analysis. J Strength Cond Res. (2000) 14:170–174. Available from: https://journals.lww.com/nsca-jscr/Fulltext/2000/05000/Age_Related_Aerobic_Power_in_Volunteer.9.aspx

[11] Elsner KL Kolkhorst FW. Metabolic demands of simulated firefighting tasks. Ergonomics. (2008) 51:1418–25. doi: 10.1080/00140130802120259, PMID: 18802822

[12] Ras J Kengne AP Smith DL Soteriades ES November RV Leach L. Effects of cardiovascular disease risk factors, musculoskeletal health, and physical fitness on occupational performance in firefighters: a systematic review and meta-analysis. Int J Environ Res Public Health. (2022) 19:11946. doi: 10.3390/ijerph191911946, PMID: 36231242 PMC9564707

[13] Rhea MR Alvar BA Gray R. Physical fitness and job performance of firefighters. J Strength Cond Res. (2004) 18:348–52. doi: 10.1519/R-12812.1, PMID: 15142006

[14] Skinner TL Kelly VG Boytar AN Peeters G Rynne SB. Aviation rescue firefighters physical fitness and predictors of task performance. J Sci Med Sport. (2020) 23:1228–33. doi: 10.1016/j.jsams.2020.05.013, PMID: 32507623

[15] Smith DL Barr DA Kales SN. Extreme sacrifice: sudden cardiac death in the US fire service. Extrem Physiol Med. (2013) 2:1–9. doi: 10.1186/2046-7648-2-6 23849605 PMC3710100

[16] Yang J Teehan D Farioli A Baur DM Smith D Kales SN. Sudden cardiac death among firefighters ≤45 years of age in the United States. Am J Cardiol. (2013) 112:1962–7. doi: 10.1016/j.amjcard.2013.08.029, PMID: 24079519 PMC7750027

[17] Sen S Palmieri T Greenhalgh D. Cardiac fatalities in firefighters: an analysis of the U.S. fire administration database. J Burn Care Res. (2016) 37:191–5. doi: 10.1097/BCR.0000000000000225, PMID: 25501775

[18] Ras J Leach L. Prevalence of coronary artery disease risk factors in firefighters in the city of Cape Town fire and rescue service – a descriptive study. J Public Health Res. (2021) 10:2000. doi: 10.4081/jphr.2021.2000 33623778 PMC7887455

[19] Ras J Leach L. Firefighters’ health knowledge, cardiovascular disease risk factors and sociodemographic characteristics as predictors of firefighters attitudes toward health. J Occup Environ Med. (2022) 64:e705–13. doi: 10.1097/JOM.0000000000002679, PMID: 35973044

[20] Ras J Mosie D Strauss M Leach L. Knowledge of and attitudes toward health and cardiovascular disease risk factors among firefighters in Cape Town, South Africa. J Public Health Res. (2021) 11:2307. doi: 10.4081/jphr.2021.2307 34351095 PMC8859729

[21] Bucala M Sweet E. Obesity in the fire service: an inside look at the perceptions of firefighters towards obesity and other health issues. Res Sq. (2019). doi: 10.21203/rs.2.15518/v1

[22] Dobson M Choi B Schnall PL Wigger E Garcia-Rivas J Israel L. Exploring occupational and health behavioral causes of firefighter obesity: a qualitative study. Am J Ind Med. (2013) 56:776–90. doi: 10.1002/ajim.22151, PMID: 23335437

[23] Kay BF Lund MM Taylor PN Herbold NH. Assessment of firefighters’ cardiovascular disease-related knowledge and behaviors. J Am Diet Assoc. (2001) 101:807–9. doi: 10.1016/S0002-8223(01)00200-0, PMID: 11478481

[24] Hong O Phelps S Feld J Vogel S. Occupational injuries, duty status, and factors associated with injuries among firefighters. Workplace Health Saf. (2012) 60:517–23. doi: 10.1177/216507991206001203, PMID: 23163314

[25] Yoon JH Kim YK Kim KS Ahn YS. Characteristics of workplace injuries among nineteen thousand Korean firefighters. J Korean Med Sci. (2016) 31:1546. doi: 10.3346/jkms.2016.31.10.1546, PMID: 27550481 PMC4999395

[26] Nazari G Osifeso TA MacDermid JC. Distribution of number, location of pain and comorbidities, and determinants of work limitations among firefighters. Rehabil Res Practice. (2020), 2020:1942513 10.1155/2020/1942513 PMC7669334 33224531

[27] Durand G Tsismenakis AJ Jahnke SA Baur DM Christophi CA Kales SN. Firefighters’ physical activity: relation to fitness and cardiovascular disease risk. Med Sci Sports Exerc. (2011) 43:1752–9. doi: 10.1249/MSS.0b013e318215cf25, PMID: 21364484

[28] Barry AM Lyman KJ Dicks ND Landin KD McGeorge CR Hackney KJ. Firefighters’ physical activity and waist circumference as predictors of VO2max. J Occup Environ Med. (2019) 61:849–53. doi: 10.1097/JOM.0000000000001690, PMID: 31393276

[29] Yu CCW Au CT Lee FYF So RCH Wong JPS Mak GYK. Association between leisure time physical activity, cardiopulmonary fitness, cardiovascular risk factors, and cardiovascular workload at work in firefighters. Saf Health Work. (2015) 6:192–9. doi: 10.1016/j.shaw.2015.02.004, PMID: 26929827 PMC4674482

[30] Ras J Leach L. Relationship between physical activity, coronary artery disease risk factors and musculoskeletal injuries in the City of Cape Town fire and rescue service. INQUIRY: J Health Care Organ Provis Financ. (2022) 59:00469580221084485. doi: 10.1177/00469580221084485 PMC8958692 35341350

[31] Vicente MM Herrero DC Prieto JP. Cardiorespiratory fitness in Spanish firefighters. J Occup Environ Med. (2021) 63:e318–22. doi: 10.1097/JOM.0000000000002199, PMID: 33769332

[32] Baur DM Christophi CA Cook EF Kales SN. Age-related decline in cardiorespiratory fitness among career firefighters: modification by physical activity and adiposity. J Obes. (2012) 2012:10903. doi: 10.1155/2012/710903, PMID: 22666557 PMC3361265

[33] Ras J Soteriades ES Smith DL Kengne AP Leach L. Association between physical fitness and musculoskeletal health in firefighters. Front Physiol. (2023):14. doi: 10.3389/fphys.2023.1210107 PMC10352848 37469568

[34] Poston WSC Jitnarin N Haddock CK Jahnke SA Tuley BC. Obesity and injury-related absenteeism in a population-based firefighter cohort. Obesity. (2011) 19:2076–81. doi: 10.1038/oby.2011.147, PMID: 21633400 PMC5831189

[35] Phelps SM Drew-Nord DC Neitzel RL Wallhagen MI Bates MN Hong OS. Characteristics and predictors of occupational injury among career firefighters. Workplace Health Saf. (2018) 66:291–301. doi: 10.1177/2165079917740595, PMID: 29251258 PMC6097705

[36] Williford HN Duey WJ Olson MS Howard R Wang N. Relationship between fire fighting suppression tasks and physical fitness. Ergonomics. (1999) 42:1179–86. doi: 10.1080/001401399185063, PMID: 10503052

[37] Davis PO Dotson CO Santa Maria DL. Relationship between simulated fire fighting tasks and physical performance measures. Med Sci Sports Exerc. (1982) 14:13. doi: 10.1249/00005768-198201000-00013, PMID: 7070261

[38] von Heimburg E Ingulf Medbø J Sandsund M Reinertsen RE. Performance on a work-simulating firefighter test versus approved laboratory tests for firefighters and applicants. Int J Occup Saf Ergon. (2013) 19:227–43. doi: 10.1080/10803548.2013.11076981, PMID: 23759193

[39] Davis SC Jankovitz KZ Rein S. Physical fitness and cardiac risk factors of professional firefighters across the career span. Res Q Exerc Sport. (2002) 73:363–70. doi: 10.1080/02701367.2002.10609033, PMID: 12230346

[40] Seyedmehdi SM Attarchi M Cherati AS Hajsadeghi S Tofighi R Jamaati H. Relationship of aerobic fitness with cardiovascular risk factors in firefighters. Work. (2016) 55:155–61. doi: 10.3233/WOR-162375, PMID: 27612056

[41] Strauss M Foshag P Jehn U Brzęk A Littwitz H Leischik R. Higher cardiorespiratory fitness is strongly associated with lower cardiovascular risk factors in firefighters: a cross-sectional study in a German fire brigade. Sci Rep. (2021) 11:2445. doi: 10.1038/s41598-021-81921-1, PMID: 33510237 PMC7843993

[42] Poplin GS Roe DJ Peate W Harris RB Burgess JL. The Association of Aerobic Fitness with Injuries in the fire service. Am J Epidemiol. (2014) 179:149–55. doi: 10.1093/aje/kwt213 24186973

[43] Ras J Smith DL Soteriades ES Kengne AP Leach L. A pilot study on the relationship between cardiovascular health, musculoskeletal health, physical fitness and occupational performance in firefighters. Eur J Investig Health Psychol Educ. (2022) 12:1703–18. doi: 10.3390/ejihpe12110120, PMID: 36421326 PMC9689559

[44] Liguori G Medicine AC of S, Fountaine CJ. ACSM’s guidelines for exercise testing and prescription. Wolters Kluwer; (2021). (American College of Sports Medicine Series). Available from: https://books.google.co.za/books?id=6P-azQEACAAJ

[45] Rexhepi AM Brestovci B. Prediction of VO2max based on age, body mass, and resting heart rate. Human Movement. (2014) 15:56–9. doi: 10.2478/humo-2014-0003

[46] D’Agostino RB Vasan RS Pencina MJ Wolf PA Cobain M Massaro JM. General cardiovascular risk profile for use in primary care. Circulation. (2008) 117:743–53. doi: 10.1161/CIRCULATIONAHA.107.699579, PMID: 18212285

[47] Lloyd-Jones DM Leip EP Larson MG D’Agostino RB Beiser A Wilson PWF. Prediction of lifetime risk for cardiovascular disease by risk factor burden at 50 years of age. Circulation. (2006) 113:791–8. doi: 10.1161/CIRCULATIONAHA.105.548206, PMID: 16461820

[48] Lloyd-Jones DM Braun LT Ndumele CE Smith SC Sperling LS Virani SS. Use of risk assessment tools to guide decision-making in the primary prevention of atherosclerotic cardiovascular disease: a special report from the American Heart Association and American College of Cardiology. Circulation. (2019) 139:e1162–77. doi: 10.1161/CIR.0000000000000638, PMID: 30586766

[49] Hedge A Morimoto S Mccrobie D. Effects of keyboard tray geometry on upper body posture and comfort. Ergonomics. (1999) 42:1333–49. doi: 10.1080/001401399184983 10582503

[50] Crawford JO. The Nordic musculoskeletal questionnaire. Occup Med (Chic Ill). (2007) 57:300–1. doi: 10.1093/occmed/kqm036, PMID: 37474194

[51] Templeton GF. A two-step approach for transforming continuous variables to normal: implications and recommendations for IS research. Commun Assoc Inf Syst. (2011) 28:41–58. doi: 10.17705/1CAIS.02804

[52] Siddall AG Stevenson RDM Turner PJF Bilzon JLJ. Physical and physiological performance determinants of a firefighting simulation test. J Occup Environ Med. (2018) 60:637–43. doi: 10.1097/JOM.0000000000001313, PMID: 29485491

[53] Saari AI Renz G Davis P Abel MG. The influence of age on firefighter combat challenge performance and exercise training habits. J. Strength Cond. Res. (2020). Available at: www.nsca.com 10.1519/JSC.0000000000003714 32604151

[54] Siddall AG Stevenson RDM Turner PFJ Stokes KA Bilzon JLJ Stevenson RDM. Development of role-related minimum cardiorespiratory fitness standards for firefighters and commanders (2016) 59:1335. doi: 10.1080/00140139.2015.1135997, 26853098

[55] Phillips DB Scarlett MP Petersen SR. The influence of body mass on physical fitness test performance in male firefighter applicants. J Occup Environ Med. (2017) 59:1101–8. doi: 10.1097/JOM.0000000000001145, PMID: 29116989

[56] Perroni F Guidetti L Cignitti L Baldari C. Age-related changes in upper body strength and lower limb power of professional Italian firefighters. Sport Sci. Health. (2015) 11:279–85. doi: 10.1007/s11332-015-0236-y

[57] Perroni F Cignitti L Cortis C Capranica L. Physical fitness profile of professional Italian firefighters: differences among age groups. Appl Ergon. (2014) 45:456–61. doi: 10.1016/j.apergo.2013.06.005, PMID: 23849328

[58] Walker A Driller M Argus C Cooke J Rattray B. The ageing Australian firefighter: An argument for age-based recruitment and fitness standards for urban fire services. 57, Ergonomics. Taylor & Francis; (2014), 612–621 10.1080/00140139.2014.887790 24588283

[59] Chu DJ al Rifai M Virani SS Brawner CA Nasir K Al-Mallah MH. The relationship between cardiorespiratory fitness, cardiovascular risk factors and atherosclerosis. Atherosclerosis. (2020) 304:44–52. doi: 10.1016/j.atherosclerosis.2020.04.019, PMID: 32590246

[60] Donovan R Nelson T Peel J Lipsey T Voyles W Israel RG. Cardiorespiratory fitness and the metabolic syndrome in firefighters. Occup Med (Chic Ill). (2009) 59:487–92. doi: 10.1093/occmed/kqp095, PMID: 19578075

[61] Myhre LG Tucker DM Bauer DH Fisher JR Jr Grimm WH. Relationship between selected measures of physical fitness and performance of a simulated fire fighting emergency task. ARMSTRONG LAB BROOKS AFB TX; (1997). Available at: https://apps.dtic.mil/sti/citations/ADA319915

[62] Schonfeld BR Doerr DF Convertino VA. An occupational performance test validation program for fire fighters at the Kennedy Space Center. J Occup Environ Med. (1990) 32. doi: 10.1097/00043764-199007000-00016, PMID: 2391579

[63] Siddall A Standage M Stokes K Bilzon J. Development of occupational fitness standards for the UK fire and rescue services. (FRS: University of Bath) (2014) (October). 59:1–59.

[64] Nazari G Lu S MacDermid JC. Quantifying physiological responses during simulated tasks among Canadian firefighters: a systematic review and meta-analysis. J Mil Veteran Fam Health. (2021) 7:55–75. doi: 10.3138/jmvfh-2019-0063

[65] Nazari G MacDermid JC Sinden KE Overend TJ. The relationship between physical fitness and simulated firefighting task performance. Rehabil Res Pract. (2018) 2018:1–7. doi: 10.1155/2018/3234176, PMID: 29850253 PMC5924985

[66] Misner JE Boileau RA Plowman SA Elmore BG Gates MA Gilbert JA. Leg power characteristics of female firefighter applicants. J Occup Environ Med. (1988) 30:433–7. doi: 10.1097/00043764-198805000-00011 3373348

[67] Kleinberg CR Ryan ED Tweedell AJ Barnette TJ Wagoner CW. Influence of lower extremity muscle size and quality on stair-climb performance in career firefighters. J Strength Cond Res. (2016) 30:1613–8. doi: 10.1519/JSC.0000000000001268, PMID: 26605810

[68] Hilyer JC Brown KC Sirles AT Peoples L. A flexibility intervention to reduce the incidence and severity of joint injuries among municipal firefighters. J Occup Med. (1990) 32:631–7. doi: 10.1097/00043764-199007000-00015, PMID: 2391578

[69] DeFina LF Haskell WL Willis BL Barlow CE Finley CE Levine BD. Physical activity versus cardiorespiratory fitness: two (partly) distinct components of cardiovascular health? Prog Cardiovasc Dis. (2015) 57:324–9. doi: 10.1016/j.pcad.2014.09.008, PMID: 25269066

[70] Alves AJ Viana JL Cavalcante SL Oliveira NL Duarte JA Mota J. Physical activity in primary and secondary prevention of cardiovascular disease: overview updated. World J Cardiol. (2016) 8:575–83. doi: 10.4330/wjc.v8.i10.575, PMID: 27847558 PMC5088363

[71] Hauschild VD DeGroot DW Hall SM Grier TL Deaver KD Hauret KG, Occup environ med. 74, Occupational and Environmental Medicine. BMJ Publishing Group; (2017). 144–153. 27810940 10.1136/oemed-2016-103684

[72] Lesniak AY Sell KM Morris C Abel MG. Relationship between heart rate variability vs. occupational performance, physical activity and fitness measures in structural firefighters. J Sport Human Perf. (2022) 10:56–72. doi: 10.12922/jshp.v10i1.169

[73] Porto LGG Schmidt ACB de Souza JM Nogueira RM Fontana KE Molina GE. Firefighters’ basal cardiac autonomic function and its associations with cardiorespiratory fitness. Work. (2019) 62:485–95. doi: 10.3233/WOR-192883, PMID: 30909264

[74] Tomes C Schram B Orr R. Relationships between heart rate variability, occupational performance, and fitness for tactical personnel: a systematic review. Front Public Health. (2020) 8:583336. doi: 10.3389/fpubh.2020.583336 33240835 PMC7680786

[75] Rodrigues S Paiva JS Dias D Cunha JPS. Stress among on-duty firefighters: an ambulatory assessment study. PeerJ. (2018) 6:e5967. doi: 10.7717/peerj.5967, PMID: 30581658 PMC6294048

[76] Ras J Leach L. Use of Mobile Technology in Assessing Occupational Performance and Stress in firefighters. Mentor D, (Ed.) Handbook of research on new media, training, and skill development for the modern workforce. Hershey, PA, USA: IGI Global; (2022), 150–186

[77] Williams-Bell FM Villar R Sharratt MT Hughson RL. Physiological demands of the firefighter candidate physical ability test. Med Sci Sports Exerc. (2009) 41:653–62. doi: 10.1249/MSS.0b013e31818ad117, PMID: 19204584

[78] MacDermid JC Tang K Sinden KE D’Amico R. Work functioning among firefighters: a comparison between self-reported limitations and functional task performance. J Occup Rehabil. (2019) 29:194–204. doi: 10.1007/s10926-018-9778-6, PMID: 29802581

[79] Muegge CM Zollinger TW Song Y Wessel J Monahan PO Moffatt SM. Barriers to weight management among overweight and obese firefighters. J Occup Environ Med. (2020) 62:37–45. doi: 10.1097/JOM.0000000000001751, PMID: 31651603

[80] Ras J Smith DL Soteriades ES Kengne AP Leach L. Association between physical fitness and cardiovascular health in firefighters. Int J Environ Res Public Health. (2023) 20. doi: 10.3390/ijerph20115930, PMID: 37297534 PMC10252711

[81] Smith DL Haller JM Korre M Fehling PC Sampani K Grossi Porto LG. Pathoanatomic findings associated with duty-related cardiac death in US firefighters: a case-control study. J Am Heart Assoc. (2018) 7:e009446. doi: 10.1161/JAHA.118.009446, PMID: 30371185 PMC6222959

