# Supplementary material for: Physical fitness, cardiovascular and musculoskeletal health, and occupational performance in firefighters
Source: Front Public Health. 2023 Aug 25;11:1241250. doi: 10.3389/fpubh.2023.1241250 (PMC10485367; doi:10.3389/fpubh.2023.1241250)
Supplement: Supplementary file 1 [file Table_1.DOCX]

Supplementary Table 1: Backward multiple linear regression models to explore the association between physical fitness and cardiovascular health in physical ability test performance.

|  | Variable | R^2^ | B | SE | β | VIF |
| --- | --- | --- | --- | --- | --- | --- |
| Model: Physical fitness |  | 0.490 ^§^ |  | 125.96 |  |  |
|  | abV̇O_2max_ |  | -102.85 | 34.08 | -0.155 | 1.36 |
|  | Grip strength |  | -1.70 | 0.54 | -.169 | 1.60 |
|  | Leg strength |  | -0.67 | 0.36 | -.108 | 1.71 |
|  | Push-ups |  | -2.64 | 0.81 | -0.207 | 2.07 |
|  | Sit-ups |  | -3.53 | 1.03 | -0.207 | 1.89 |
|  | Lean body mass |  | -5.19 | 1.05 | -0.286 | 1.73 |
|  | Intercept |  | 1566.97 | 110.77 |  |  |
| Model: Cardiovascular health |  | 0.301 ^§^ |  | 149.17 |  |  |
|  | Age |  | 3.99 | 1.01 | 0.229 | 1.22 |
|  | Body mass index |  | -12.90 | 2.26 | -0.321 | 2.56 |
|  | Body fat percentage |  | 8.34 | 1.48 | 0.473 | 2.29 |
|  | Systolic blood pressure |  | 3.10 | 0.83 | -0.267 | 1.87 |
|  | Diastolic blood pressure |  | 3.69 | 1.11 | 0.240 | 1.92 |
|  | Weekly MET minutes |  | -0.01 | 0.00 | -0.238 | 1.04 |
|  | Intercept |  | 590.02 | 93.29 |  |  |
| Model: Physical fitness and cardiovascular health |  | 0.505 ^§^ |  | 123.34 |  |  |
|  | Weekly MET minutes |  | -0.01 | 0.00 | -0.136 | 1.11 |
|  | Bodyfat percentage |  | 4.80 | 0.95 | 0.256 | 1.35 |
|  | abV̇O_2max_ |  | -199.87 | 33.95 | -0.304 | 1.41 |
|  | Grip strength |  | -2.40 | 0.54 | -0.239 | 1.55 |
|  | Leg strength |  | -0.85 | 0.34 | -0.140 | 1.68 |
|  | Sit-ups |  | -3.24 | 0.83 | -0.190 | 1.25 |
|  | Intercept |  | 1426.22 | 100.70 |  |  |

**Note:** § − indicates statistical significance <0.001; B − unstandardized beta coefficient; SE – standard error; β − standardized beta coefficient; R^2^ − R squared; VIF − variation inflation factor.
